# Effect of Familiarity on Reward Anticipation in Children with and without Autism Spectrum Disorders

**DOI:** 10.1371/journal.pone.0106667

**Published:** 2014-09-03

**Authors:** Katherine K. M. Stavropoulos, Leslie J. Carver

**Affiliations:** Psychology Department, University of California San Diego, La Jolla, California, United States of America; UNC Chapel Hill, United States of America

## Abstract

**Background:**

Previous research on the reward system in autism spectrum disorders (ASD) suggests that children with ASD anticipate and process social rewards differently than typically developing (TD) children—but has focused on the reward value of unfamiliar face stimuli. Children with ASD process faces differently than their TD peers. Previous research has focused on face processing of unfamiliar faces, but less is known about how children with ASD process familiar faces. The current study investigated how children with ASD anticipate rewards accompanied by familiar versus unfamiliar faces.

**Methods:**

The stimulus preceding negativity (SPN) of the event-related potential (ERP) was utilized to measure reward anticipation. Participants were 6- to 10-year-olds with (*N* = 14) and without (*N* = 14) ASD. Children were presented with rewards accompanied by incidental face or non-face stimuli that were either familiar (caregivers) or unfamiliar. All non-face stimuli were composed of scrambled face elements in the shape of arrows, controlling for visual properties.

**Results:**

No significant differences between familiar versus unfamiliar faces were found for either group. When collapsing across familiarity, TD children showed larger reward anticipation to face versus non-face stimuli, whereas children with ASD did not show differential responses to these stimulus types. Magnitude of reward anticipation to faces was significantly correlated with behavioral measures of social impairment in the ASD group.

**Conclusions:**

The findings do not provide evidence for differential reward anticipation for familiar versus unfamiliar face stimuli in children with or without ASD. These findings replicate previous work suggesting that TD children anticipate rewards accompanied by social stimuli more than rewards accompanied by non-social stimuli. The results do not support the idea that familiarity normalizes reward anticipation in children with ASD. Our findings also suggest that magnitude of reward anticipation to faces is correlated with levels of social impairment for children with ASD.

## Introduction

Autism spectrum disorder (ASD) is a disorder defined by social-communicative deficits and repetitive and restricted behaviors. ASD is estimated to effect up to 1 in 68 children in the US (Centers for Disease Control and Prevention [CDC], 2014). Children with ASD have well documented difficulties in multiple aspects of social communication, including eye contact [1], [2], language [3], and joint attention [1], in addition to having repetitive behaviors and restricted interests.

Several theories have emerged concerning why individuals with ASD are impaired relative to their neurotypical peers in social abilities. One is the social motivation hypothesis [4]–[9]. According to this idea, children with ASD are less intrinsically motivated to attend to and engage with others, which leads to downstream social deficits. The social motivation hypothesis might predict, then, that children with ASD need to be more motivated than TD children in order to find faces rewarding. In the current study, we tested the hypothesis that, although unfamiliar faces may not be rewarding for children with ASD, a socially important familiar face, such a caregiver's face, may have greater reward value than an unfamiliar face.

There is reason to believe that children with autism might respond differently to a caregiver's face than to other, unfamiliar faces. Previous literature has investigated how children with and without ASD react to their caregivers, and whether attachment relationships differ between the two groups. The attachment literature suggests that children with ASD show somewhat typical and secure attachment relationships to their caregivers [10], [11], although a recent meta-analysis suggested that children with ASD are less likely to be securely attached compared to TD children and those with other developmental disorders [12]. Given the suggestion that children with ASD may react to their parents similarly to TD children despite their social impairments, it is possible that familiar faces may be particularly salient to children with ASD, and may “normalize” the neural responses of people with ASD [13]. While this is an intriguing possibility, no prior study has directly investigated the effect of face familiarity on the brain's reward system in ASD. The current study was designed to investigate whether familiar faces would increase reward anticipation in children with ASD compared to their TD peers.

Previous literature has documented different neural responses in individuals with ASD compared to their TD peers when viewing unfamiliar faces [14]–[16]. The relatively small literature on the effect of familiarity in ASD has been limited to the effect of familiarity on face processing [13], [17]–[25]. The studies on familiarity have varied results, likely due to inter-study differences in participants' age, methodologies, and stimuli. Previous literature on the reward system in ASD has also had mixed results, with some studies finding reward deficits in social rewards only, and others finding global reward deficits. One recent study has integrated these two lines of research and investigated familiar versus unfamiliar faces, as well as monetary rewards in a behavioral paradigm and found that both face and monetary rewards improved behavioral performance for individuals with and without ASD in a go/no-go task [26]. In order to setup and motivate the current study, we next briefly review the research on the reward system in ASD individuals using electrophysiology, functional neuroimaging, and combined methodologies, and then review previous research on the effect of familiar faces in ASD.

### Reward System in ASD

#### Electrophysiological studies

Event-related potentials (ERP) are brain potentials recorded at the surface of the scalp. These recordings reflect synchronous firing of groups of synapses, and have been used to measure the time course of brain activity related to the anticipation or processing of specific discrete events.

ERPs have been used to study the reward system in ASD. Three studies have compared reward anticipation between TD individuals and those with ASD [27]–[29]. One study used a probabilistic learning task with monetary rewards and found that children with ASD and ADHD demonstrated larger neural responses than TD children when anticipating positive outcomes, but equivalent responses when anticipating negative outcomes [27]. A second study measured attentional ERP components in response to cues triggering trials with social vs. nonsocial rewards and found that TD children exhibited larger attentional components during reward versus non-reward conditions, but children with autism did not. In addition, children with autism exhibited smaller attentional components after cues initiating social reward anticipation trials [28]. A third study measured neural correlates of reward anticipation in a guessing game task with social and nonsocial rewards and found group differences such that children with ASD showed reduced brain activity when anticipating rewards accompanied by intact versus scrambled faces [29]. Taken together, ERP studies of social reward anticipation provide evidence that individuals with ASD elicit less brain activity when anticipating social rewards compared to their TD peers.

Previous ERP studies have also investigated electrophysiological correlates of reward processing in ASD. In studies examining reward processing in ASD, two studies have utilized a guessing game with monetary rewards. Both studies found similar activation patterns in children with ASD and TD [30], [31], suggesting that children with ASD do not demonstrate deficits in reward feedback processing when the rewards are monetary. Our previous investigation of social versus non-social rewards revealed group differences in reward processing between TD children and those with ASD—especially for social stimuli [29].

#### Functional neuroimaging studies

Previous research on social versus nonsocial rewards in ASD has also utilized functional magnetic resonance imaging (fMRI). The fMRI literature on social versus nonsocial rewards in ASD vs. TD is mixed. Some studies have suggested that individuals with ASD may elicit reduced neural activation for monetary rewards compared to TD children, but have similar neural activation for social rewards [32]; others have found reduced brain activity in response to social rewards in ASD [33].

#### Behavioral studies

One recent study has investigated reward responsiveness to both familiar versus unfamiliar faces, as well as nonsocial rewards, in both TD children and those with ASD using a modified go/no-go task [26]. Children either received auditory or visual indicators of reward after successful response inhibition. The authors found that both monetary and social (both familiar and unfamiliar faces) rewards increased performance versus a control (no-reward) condition. The authors did not find evidence of decreased responsiveness to social rewards in children with ASD, but found that parents' practices with rewards and contingencies at home strongly predicted performance in the ASD group [26].

### Effects of Familiarity in ASD

#### Electrophysiological studies

We now turn to previous research investigating the effects of familiarity on face processing in ASD. Several ERP studies have measured responses to familiar and unfamiliar faces. Some investigations have found that individuals with ASD are less responsive to familiar faces compared to their typically developing peers [18], [25], yet others have found that responsiveness to familiarity may be typical, but delayed, in ASD [24], or may increase after exposure to social skills groups [17]. Conversely, other investigations found no differences between adults with and without ASD in responsiveness to familiar faces [23], or in children at high versus low risk for ASD [20], [34]. The ERP literature on the effects of familiarity on face processing in ASD is widely varied, and likely depends on a variety of factors, including cognitive functioning, age of participants, and the tasks utilized.

#### Functional Neuroimaging Studies

Two studies have investigated recognition of face familiarity using functional neuroimaging with individuals with and without ASD [13], [22]. In a study of adults, both typical and ASD groups showed increased neural activation in response to familiar versus unfamiliar faces. [22]. In a study of school-aged children with and without ASD, children with ASD demonstrated similar brain activity to their TD peers when viewing pictures of children or familiar adults, but reduced activation when viewing pictures of unfamiliar adults [13]. In contrast to these findings, many studies in which brain responses are elicited to novel faces suggest that people with ASD do not activate face-processing brain areas to the same degree that TD controls do [16], [35]. Thus, the results of recent face processing studies that have manipulated familiarity using fMRI measures suggest that brain responses might be normalized when familiar faces are used as stimuli.

### Summary

Previous research on the *reward* system in ASD has been mixed, likely due to the wide variety of methodologies and procedures utilized. However, several studies have found that individuals with ASD have differences in the neural correlates of the reward system compared to TD individuals. Similarly, previous investigations of *familiar faces* on face processing have met with mixed findings. While previous literature has investigated the effects of familiar faces on face processing, as well as the effects of social versus nonsocial stimuli on the reward system in ASD, only one study has directly investigated the effect of familiar faces on reward responsiveness in ASD [26]. No previous studies have investigated the effects of familiarity on *neural correlates* of reward in TD versus ASD.

### Current Study

The aim of the current study was to utilize electrophysiology to investigate the effect of familiarity on reward anticipation in response to faces versus non-faces in children with and without ASD. While previous studies have investigated the effects of familiarity on face processing, none have directly explored how the neural reward system is affected by familiarity in ASD. Specifically, we wanted to investigate reward anticipation for familiar versus unfamiliar faces, and scrambled versions of those images.

Previous investigations using electrophysiology to measure reward anticipation focused on the stimulus preceding negativity (SPN) component [29], [36], [37] The SPN is a component of the ERP that reflects brain activity occurring before expected feedback about one's performance [38]. SPN reflects the *expectation* of reward, and related activity of the dopaminergic reward system [39]. Our previous study of the SPN in children with ASD versus their TD peers revealed differences in how children with ASD anticipate social stimuli (pictures of faces) [29]. However, this previous study utilized a variety of unfamiliar faces.

The current study utilized one familiar and one unfamiliar face in order to determine whether familiar faces accompanying reward stimuli normalized reward anticipation in children with ASD. This design allowed us to gain information about both the effect of familiar faces on reward anticipation, and also whether the use of only one face in each condition may lead to habituation effects over time. In the current study, we also investigated whether brain activity and behavioral measures of ASD (via the SRS-2) were correlated, and whether children with more severe social impairments had reduced reward anticipation for face stimuli. We hypothesized that TD children would have an increased SPN response to face versus arrow stimuli—and that this effect would be most pronounced for familiar versus unfamiliar faces. We hypothesized that children with ASD would not have increased SPN responses to face versus arrow stimuli overall, but would have larger SPN responses to a familiar versus unfamiliar face. Lastly, we hypothesized that we would find a specific brain-behavior correlation—children with more severe social impairments (as measured with the SRS-2) would have decreased SPN amplitude to faces.

## Methods

### Participants

To estimate the needed sample size for the current study, we ran a power analysis on data from our previous study which used the same paradigm [29]. The resulting power value of .86 yielded a sample size of 26. Therefore, we recruited 28 participants for the current study: TD children (*N* = 14) and children with ASD (*N* = 14). Each child that was tested provided an adequate number of ERP trials for analysis and was included in the final sample. Exclusionary criteria for participants with ASD included history of seizures, brain injury, neurological disorders, genetic causes of ASD (e.g. Fragile X), or any concurrent psychiatric condition (other than ASD), based on parent report. Exclusionary criteria for TD participants included all of the above criteria, plus an immediate family history of ASD. None of the children in the TD group were taking psychoactive medications. One child in the ASD group was taking medication to improve concentration, and one was taking medication to decrease aggression and stabilize mood. Participants were recruited from a UC San Diego subject pool and through postings on websites for parents of children on the autism spectrum. All participants had normal hearing and normal or corrected to normal vision. Procedures were approved by the University of California, San Diego institutional review board, and written consent was obtained from caregivers. All children over 7 years of age signed an assent form.

Table 1 provides detailed participant information. IQ scores [40] were available for all participants. TD children were matched with children with ASD on mental age (full scale IQ/100 * chronological age). No differences were found between groups on mental age, *F*(1,26) = .01. Children in the ASD group had been previously diagnosed with ASD through various sources (e.g. formal evaluations through an autism center, or school diagnosis). Diagnosis was confirmed for the current study with Module 3 of the ADOS-2 [41]. The ADOS-2 was administered by an individual trained to research reliability on administration, scoring, and interpretation of the measure.

**Table 1:** Participant characteristics including: IQ (WASI), chronological age, mental age (WASI/100 * chronological age), gender, SRS-2 T-score, and ADOS-2 severity scores for the ASD group.

Group	Participants	WASI (full-scale)	Chron. Age	Mental Age	Gender	SRS-2 SCI T-Score	SRS-2 RBB T-Score	ADOS-2 Severity Score
ASD	14	*M* = 99.42^a^ *SE* = 4.10	*M* = 8.85 *SE* = .39	*M* = 8.86 *SE* = .57	11 M 3 F	*M* = 77.50^b^ *SE* = 1.94	*M* = 80.07^c^ *SE* = 2.30	*M* = 7.14 *SE* = .46
TD	14	*M* = 112.64^a^ *SE* = 4.10	*M* = 7.94 *SE* = .39	*M* = 8.96 *SE* = .57	11 M 3 F	*M* = 43.53^b^ *SE* = 2.01	*M* = 46.38^c^ *SE* = 2.39	N/A

_a_ *p* = .03, 95% CI [−1.28 −25.14].

_b_ *p*<.0001, 95% CI [39.72 28.20].

_c_ *p*<.0001, 95% CI [40.52 26.84].

*WASI* Wechsler Abbreviated Scale of Intelligence, *SRS-2* Social Responsiveness Scale, second edition, *SCI* Social Communication and Interaction, *RBB* Restricted Interests and Repetitive Behavior, *ADOS-2* Autism Diagnostic Observation Schedule Second Edition.

### Behavioral Measures

Participants' caregivers completed the Social Responsiveness Scales (SRS-2) [42], which measures social responsiveness and behavior. We also tested for overt motivation or affective differences between groups for each condition. To accomplish this, children (*N* = 9 TD, 13 ASD) completed a 1–7 Likert rating scale of how much they enjoyed the game (1 =  “I do not like this game”, and 7 =  “I love this game”) after each block. This was used in order to gather more information about whether one group felt more or less motivated to engage in the task. Previous research suggests that the presence of reward versus no reward affects SPN amplitude—with greater SPN amplitude in reward versus no-reward conditions [43]—and we wished to assess whether both groups felt equally invested in the game. Participants also completed a 1–7 Likert scale about their perception of answering correctly (1 =  “I never got correct answers”, and 7 =  “I always got correct answers”). In reality, the correct versus incorrect answers was predetermined, equated for individuals, and controlled by experimental design; the rating was used to verify that the groups did not differ in their perception that they were obtaining correct answers.

### Stimuli and Task

The task was identical to that described in previous studies [29], [37], but the stimuli differed in order to include different blocks of trials with a familiar or an unfamiliar face. The task was a guessing game that presented blocks of trials that used left and right visual stimuli (question marks). Participants were asked to indicate their guess via button press whether the left or right stimulus was “correct.” After this choice, the left and right question marks were replaced with an arrow in the middle pointing towards whichever question mark the participant chose. This was done to reinforce the idea that participants had control over the task and their responses were being recorded.

There were four blocked feedback conditions: *familiar social*, *familiar nonsocial, unfamiliar social, and unfamiliar nonsocial*. The incidental stimulus in the familiar social condition was a picture of the child's caregiver that was smiling for “correct” answers and frowning for “incorrect” answers (photographs obtained via digital camera in our lab, and modeled after the NimStim stimulus set) [44]. The incidental stimulus in the unfamiliar social condition was a picture of another child's caregiver that was smiling for “correct” answers and frowning for “incorrect” answers. Incidental stimuli in the nonsocial conditions were composed of scrambled face elements from the social conditions formed into an arrow that pointed upwards for “correct” answers and downwards for “incorrect” answers (e.g. the stimulus in the familiar nonsocial condition was an arrow composed from the familiar social photograph, and stimulus in the unfamiliar nonsocial condition was an arrow composed from the unfamiliar social photograph). The face images and scrambled-face images were individually created from photographs taken in our lab with a digital camera. The face in the unfamiliar condition was chosen for each subject to match his or her caregiver's face on ethnicity, gender, and presence or absence of glasses. The use of scrambled faces to construct the arrow controlled for low-level visual features of the stimuli. Presented stimuli subtended a horizontal visual angle of 14.5 degrees, and a vertical visual angle of 10.67 degrees. The order in which children saw the four blocks of trials was counterbalanced between participants.

Participants were told that the reward for each correct answer was a goldfish cracker, or if they preferred, fruit snacks. They were told that if they guessed correctly, they would see a ring of intact goldfish crackers, and the goldfish would be crossed out for incorrect answers. Participants were told that the computer would sum their total of correct responses, and they would receive a goldfish cracker for each correct answer they gave, but would not lose any goldfish crackers for incorrect answers. Importantly, in both the familiar and unfamiliar social and nonsocial feedback trials, the face/arrow information was incidental. A computer program predetermined correct versus incorrect answers in pseudorandom order such that children got 50% “correct” and 50% “incorrect,” with no more than three of the same answer in a row.

The four feedback conditions were tested in separate blocks, each composed of 60 trials. There were four conditions that composed the trials (familiar face/“familiar social”; unfamiliar face/“unfamiliar social”; familiar arrow/“familiar nonsocial”; and unfamiliar arrow/ “unfamiliar nonsocial” trials). Within each block of 60 trials, there were 10-s breaks every 15 trials. During breaks, participants were asked to relax, or move if they felt restless. Between blocks, a longer break (2–5 min.) was taken. To control for attentional effects, children were observed via webcam, and trials in which they were not attending to the stimulus were marked and discarded during analysis. Of the final sample, none of the children had any trials discarded for this reason.

### EEG Recording

Participants wore a standard, fitted cap (Electrocap International) with 33 silver/silver-chloride (Ag/AgCl) electrodes placed according to the extended international 10–20 system. Continuous EEG was recorded with a NeuroScan 4.5 System with a reference electrode at Cz and re-referenced offline to the average activity at left and right mastoids. Electrode resistance was kept under 10 kOhms. Continuous EEG was amplified with a low pass filter (70 Hz), a directly coupled high pass filter (DC), and a notch filter (60 Hz). The signal was digitized at a rate of 250 samples per second via an Analog-to-Digital converter. Eye movement artifacts and blinks were monitored via horizontal electrooculogram (EOG) placed at the outer canthi of each eye and vertical EOG placed above and below the left eye. ERP trials were time locked to the onset of the feedback stimulus. The baseline period was −2200 to −2000 ms, and the data were epoched from −2200 to 100 ms. The interval between trials was varied between 1,800–2,000 ms. Trials with no behavioral response, or containing electrophysiological artifacts, were excluded from the averages.

Artifacts were removed via a four-step process. Data were visually inspected for drift exceeding +/−200 mV in all electrodes, high frequency noise visible in all electrodes larger than 100 mV, and flatlined data. Following inspection, data were epoched and eyeblink artifacts were identified using independent component analysis (ICA). Individual components were inspected alongside epoched data, and blink components were removed. To remove additional artifacts, we utilized a moving window peak-to-peak procedure in ERPlab [45], with a 200 ms moving window, a 100 ms window step, and a 150 mV voltage threshold. Participants with less than 10 artifact-free trials in any block of testing were excluded (*N* = 0). Thus, our final analysis includes 14 children with ASD and 14 TD children.

## Results

Data were analyzed using JMP (version 10.0). For our initial analysis, we separated familiarity (familiar, unfamiliar) from condition (face, arrow). We used mixed model (between and within subjects) analysis of variance (ANOVA) to test for differences between group, condition, familiarity, and caudality (anterior-posterior scalp locations).

### Behavioral Measures

As expected, SRS-2 T-scores (which reflect more severe social impairments) were significantly higher for the ASD group than the TD group for the social communication subscale *F*(1, 32) = 215, *p*<.0001, and the repetitive and restricted behavior subscale *F*(1,32) = 158.55, *p*<.0001. Means and standard deviations between groups on the SRS-2 are shown in *Table 1*. No significant differences were found between groups on children's Likert ratings of liking the game for any of the four conditions, (all *p*s>.2), or perception of generating correct answers, (all *p*s>.1)

### ERP

#### SPN

The mean amplitude of the SPN was measured between −210 and −10 ms, prior to feedback onset, as defined in previous research [29], [37], [46]. Electrode sites F3/F4, C3/C4, P3/P4, and T5/T6, which are typically maximum amplitude sites for SPN [43], were analyzed. Artifact-free trials were analyzed for each of the four conditions between groups. No significant differences were found between groups for any of the four conditions (all *p*s>.15). Mean amplitude and trial numbers for each group in all 4 conditions are shown in *Table 2*.

**Table 2:** Descriptive statistics of trial numbers and amplitude of the SPN for typically developing (TD) individuals and those with autism spectrum disorder (ASD).

Group	Familiar Faces		Unfamiliar Faces		Familiar Arrows		Unfamiliar Arrows	
	Trials	Amplitude	Trials	Amplitude	Trials	Amplitude	Trials	Amplitude
TD	30.15 (2.67)	−6.58 (2.97)	30.21 (2.72)	−3.91 (2.89)	29.92 (3.01)	−.28 (2.97)	30.14 (2.31)	−.12 (2.89)
ASD	25.07 (2.57)	−3.65 (2.89)	30.28 (2.72)	−3.74 (2.89)	28.14 (2.90)	−2.21 (2.89)	25.21 (2.31)	−5.73(2.89)

Means are displayed, followed by standard error in parentheses (SE). Amplitude is the average magnitude of the SPN over the last 200 ms before reward stimulus onset (measured in microvolts).

A 2 (Group) ×2 (Condition) ×2 (Familiarity) ×4 (Electrode location) ANOVA did not reveal a significant effect of familiarity, *F*(1, 32.06) = .23, *n.s*, or any interactions with familiarity and other variables of interest. It is possible that over the course of each block, children's response to the single repeated stimulus habituated. In order to explore this possibility, we analyzed the first and second half of each participant's accepted trials for all four blocks in a 2 (Time) ×2 (Group) 2 (Familiarity) ×2 (Condition) ×4 (Electrode location) ANOVA. There was a marginal main effect of time such that the first half of trials elicited a larger SPN than the second half, regardless of group or condition *F*(25.9) = 3.72, *p* = .064, 95% CI [−2.31 to 4.99]. No other interactions with time were significant.

**Figure 1:**
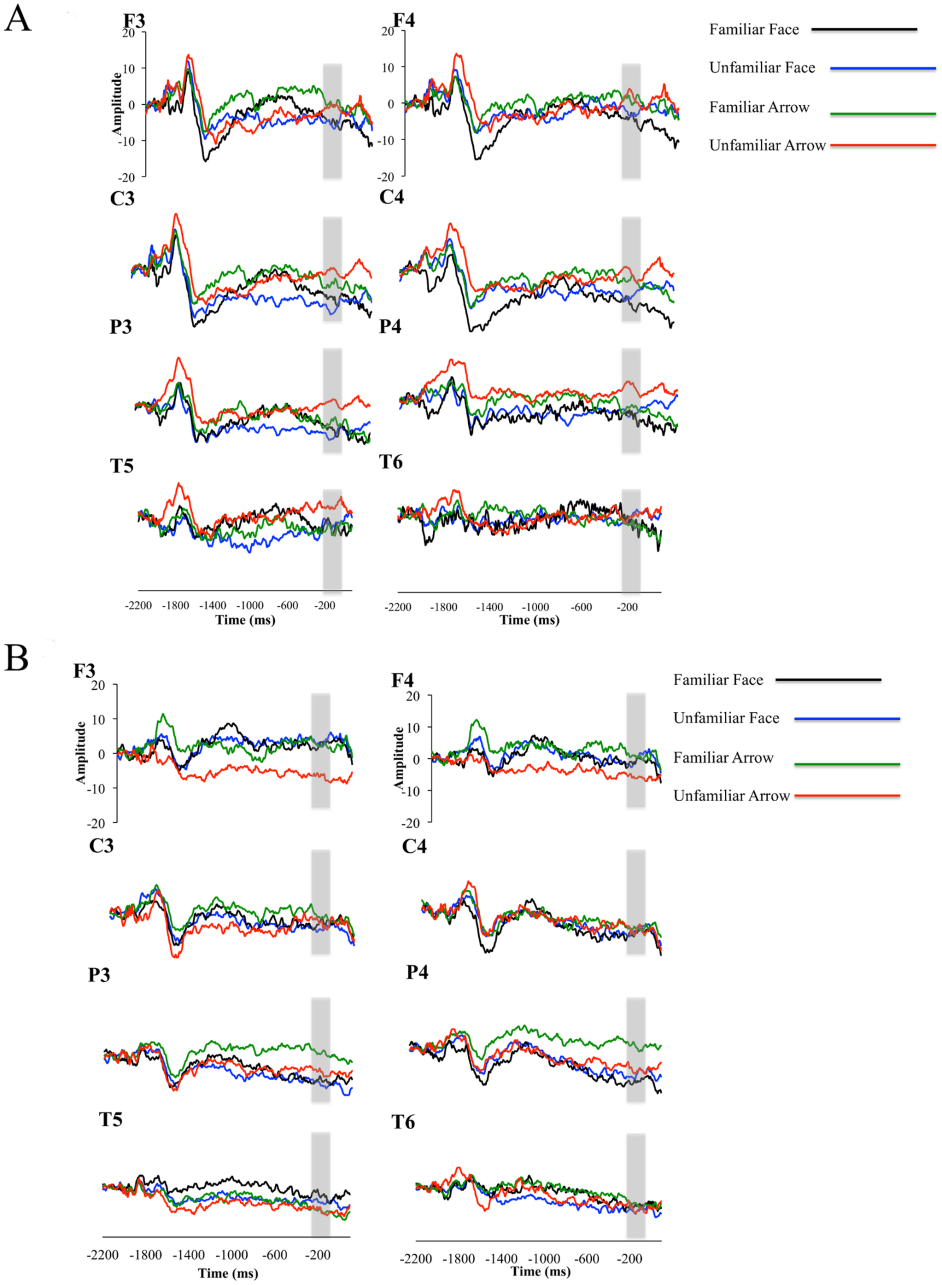
Grand averaged waveforms for the Stimulus Preceding Negativity (SPN). (A) Grand averaged waveforms for TD children from the Stimulus Preceding Negativity (SPN) prior to familiar faces, unfamiliar faces, familiar arrows, and unfamiliar arrows. (B) Grand averaged waveforms for children with ASD from the Stimulus Preceding Negativity (SPN) in ancitipation of familiar faces, unfamiliar faces, familiar arrows, and unfamiliar arrows. The area between −210 and −10 ms, used for statistical analysis, is highlighted with a grey box.

Given previous reports of differences in brain responses to familiar versus unfamiliar faces in TD children, but not those with ASD we conducted a planned 4 (Condition) ×2 (Group) ×4 (Electrode location) ANOVA for faces. We found a significant effect of group × electrode. Subsequent pairwise comparisons were non-significant. In order to better understand the effects of the different conditions on each group, a 4 (Condition) ×4 (Electrode location) ANOVA was conducted for the TD group and ASD groups separately. For TD children there was a main effect of condition, *F*(3, 37.55) = 2.76, *p* = .055, such that the familiar and unfamiliar face conditions elicited larger responses than the familiar and unfamiliar arrow conditions. Follow-up contrasts between the familiar face condition and the other three conditions (alpha corrected  = .016) revealed marginally significant differences between the familiar face condition and the unfamiliar arrow condition (*p* = .018, 95% CI [1.15 to 11.82]) as well as a marginally significant difference between the familiar face and unfamiliar arrow conditions (*p* = .02, 95% CI [.90 to 11.82]). No other pairwise comparisons were significant. For the ASD group, there was no effect of condition *F*(3, 36.24) = .53, *n.s*. *Figure 1* shows grand averages of all four conditions for each group.

**Figure 2:**
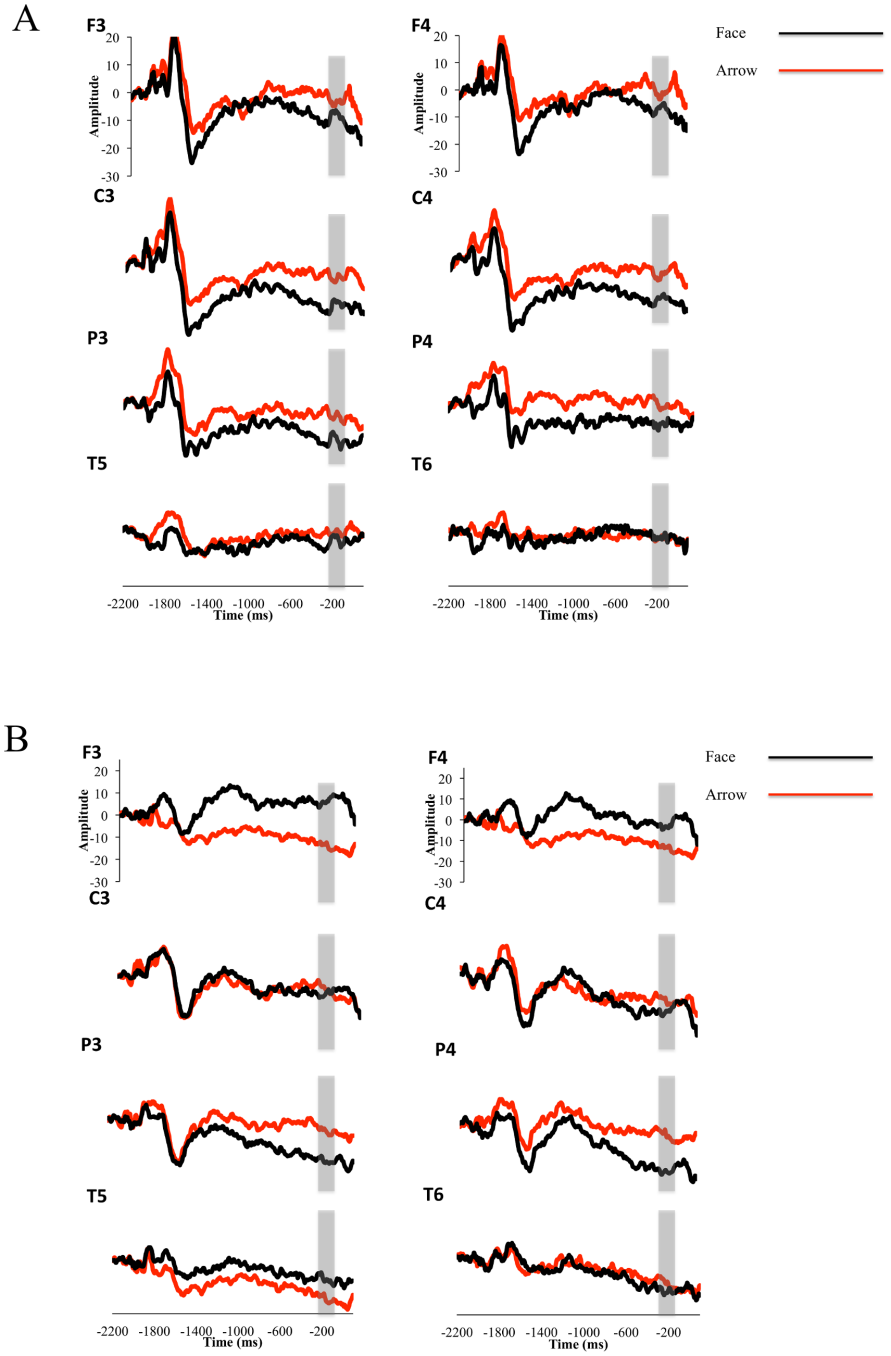
Grand averaged waveforms collapsed across familiarity. (A) Grand averaged waveforms for TD children from the Stimulus Preceding Negativity (SPN) prior to faces and arrows (collapsed across familiarity). The area between −210 and −10 ms, used for statistical analysis, is highlighted with a grey box. (B) Grand averaged waveforms for children with ASD from the Stimulus Preceding Negativity (SPN) prior to faces and arrows (collapsed across familiarity). The area between −210 and −10 ms, used for statistical analysis, is highlighted with a grey box.

Because there was no main effect of familiarity within or between groups, nor interactions involving familiarity, we collapsed across familiarity for each condition (face, arrow) separately and conducted a 2 (Group) ×2 (Condition) ×4 (Electrode location) ANOVA. This analysis resulted in a significant group × condition interaction, *F*(1, 26.03) = 5.97, *p* = .021. Pairwise comparisons (alpha corrected  = .012) revealed a significant effect of condition for the TD group, such that faces elicited a larger SPN than arrows for TD children, *F*(1, 25.75) = 8.36, *p*>.01, 95% CI [1.70 to 8.75], but not for children with ASD. *Figure 2* shows grand averages of the face and arrow conditions for each group.

There was a significant effect of electrode position, *F*(3, 77.28) = 2.72, *p* = .05, such the SPN was larger over central and parietal electrodes than frontal or temporal electrode sites. Follow-up Tukey's HSD showed that central electrode sites showed a significantly larger SPN than frontal electrode sites (*p* = .04, 95% CI [.1 to 7.67]). No other pairs of electrode sites were significantly different. There was a Condition × Electrode interaction, *F*(3, 75.59) = 2.72, *p* = .05. Pairwise comparisons (alpha corrected  = .008) revealed that the significant effect of electrode was largely driven by the face condition, *F*(3, 140.7) = 4.31, *p* = .006, such that faces elicited a larger SPN than arrows differentially over various electrode sites. Pairwise comparisons also revealed a significant effect of the parietal electrode position, *F*(1, 76.74) = 8.53, *p* = .004 95% CI [1.29 9.20], such that the face condition elicited a larger SPN than the arrow conditions at this electrode site regardless of group. There was a Group × Condition × Electrode interaction, *F*(3, 75.59) = 3.40, *p* = .02. In order to investigate the Group × Condition interaction at each electrode site, we performed contrasts at all four electrode sites. These contrasts showed a significant Group × Condition interaction (alpha corrected  = .012) at both the central, *F*(1, 78.57) = 6.51, *p* = .012, 95% CI [1.07 to 8.20], and frontal electrodes, *F*(1, 78.57) = 11.24, *p* = .001, 95% CI [2.53 to 9.66], such that for the TD group, faces elicited a larger SPN than arrows, whereas for the ASD group arrows elicited a larger SPN than faces.

#### Nc

**Figure 3:**
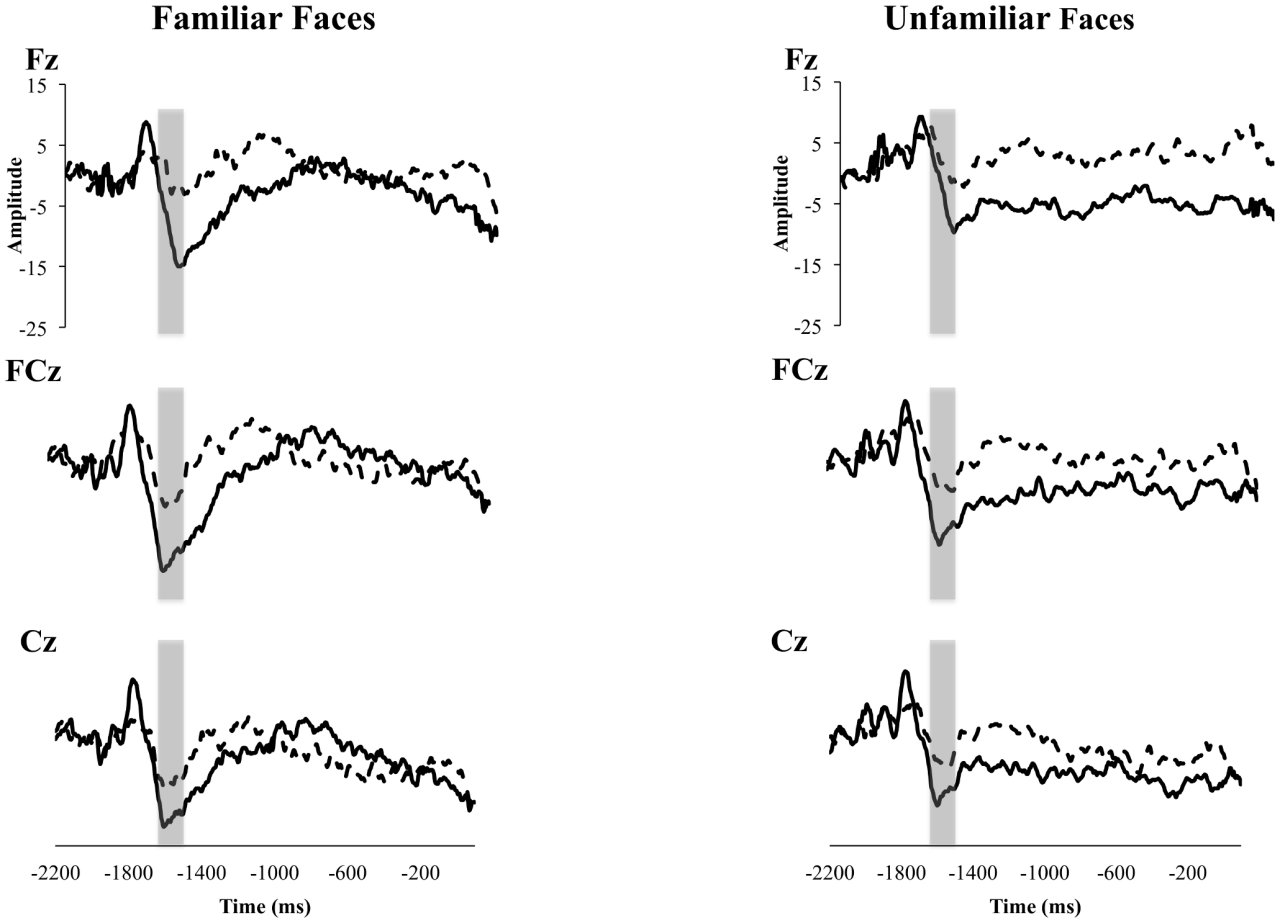
Grand averaged waveforms for both groups from the Nc component prior to familiar and unfamiliar faces. TD children are represented with a solid line, and children with ASD with a dashed line. The area between −1700 and −1550 ms, used for statistical analyses, is highlighted with a grey box.

Visual inspection of our waveforms in *Figure 1* suggested a potential difference between groups in anticipation of face stimuli in a middle latency negative component (similar to an Nc) that occurred about 400 ms after the stimulus that signaled the choice of the participant in the guessing game. The Nc is traditionally thought to reflect attention and salience in frontal and central midline electrodes, and has previously been described as a response to a presented stimulus [47], [48]. Our waveforms suggest an *anticipatory* Nc that occurred prior to the onset of face stimuli, but after children made their response. To investigate this possibility, we conducted a 2 (Group) ×2 (Familiarity) ×3 (Electrode) ANOVA for face stimuli between −1700 and −1550 ms (before the reward stimulus onset) in electrodes Fz, FCz, and Cz. Children's responses via button pad occur at −2000 ms—suggesting that this component occurred around 300 to 450 ms after the response. This time-frame (300 to 450 ms after response) is consistent with the time course of the Nc in previous investigations [47]. The ANOVA revealed a marginally significant effect of electrode, *F*(2, 52.47) = 3.10, *p* = .053. However, Tukey HSD follow-up tests did not reveal any significant differences between electrode pairs. We found a significant main effect of group, *F*(1, 26.06) = 4.91, *p* = .035, 95% CI [2.50 to 10.81], such that the face stimulus elicited a larger Nc component for TD children compared to children with ASD. No significant effects of familiarity were found, *F*(1, 25.66) = 1.8, *n.s*. We re-ran the ANOVA collapsed across familiarity and our significant effects remained. Grand averages for both groups for the face condition are seen in *Figure 3*.

### Brain-Behavior Correlations

**Figure 4:**
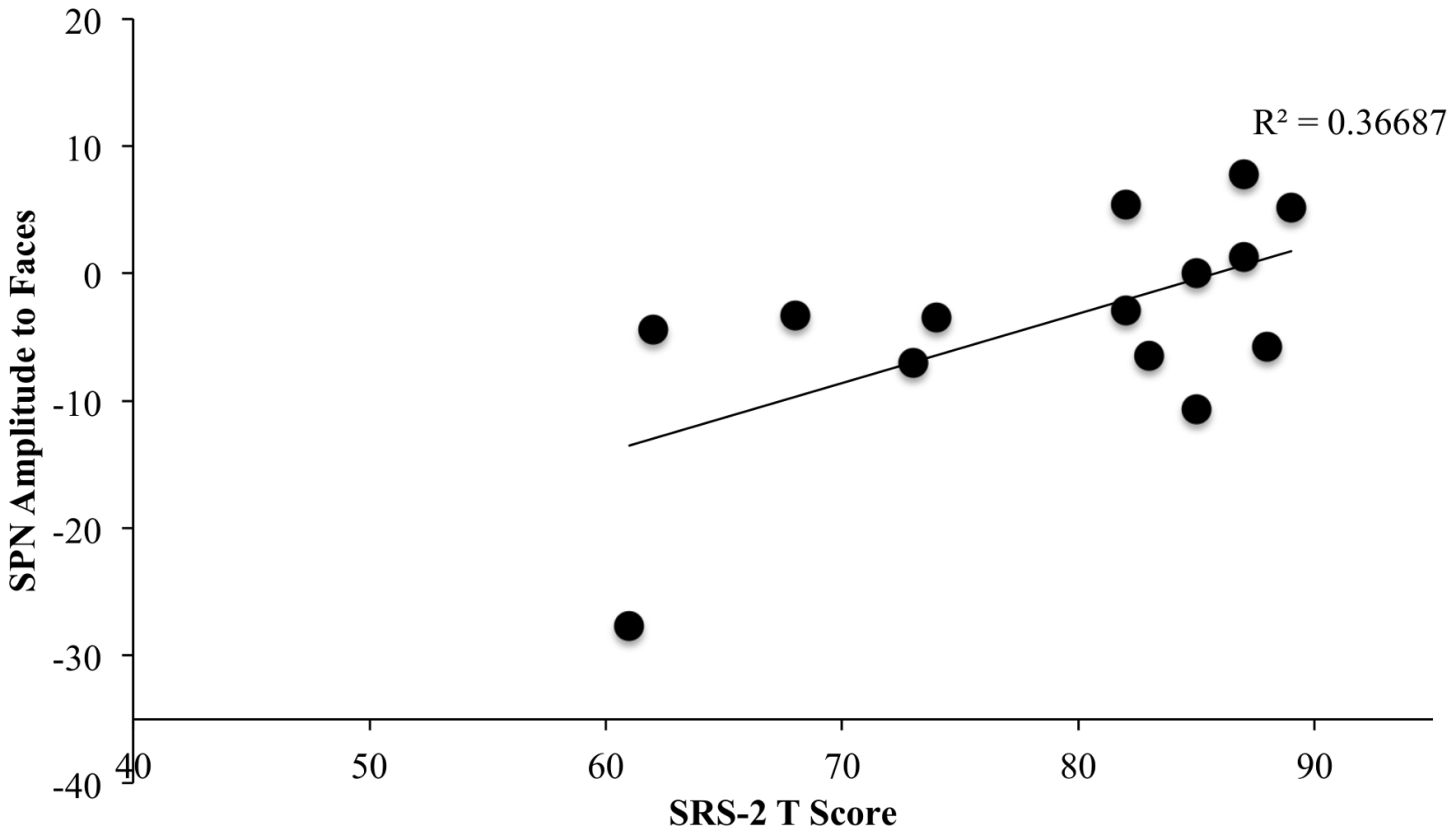
Scatter plot of SPN amplitude to faces (collapsed across familiarity) by SRS-2 T-score for children with ASD. Higher SRS-2 T-scores indicate more severe social impairments. As the SPN is a negative ERP component, more negative values indicate a larger response. Note that one participant had a particularly large SPN response and thus may be considered an outlier; and when this subject was removed, the correlation no longer reached statistical significance, *F*(1,11) = 1.5, *ns*.

We also investigated the relationship between brain activity and behavioral measures of ASD. Specifically, we asked whether magnitude of autism symptoms in the ASD group, as measured by the SRS-2, could predict the magnitude of SPN ERP response in the face condition (collapsed across familiarity). We found a significant correlation between T-scores on the SRS-2 and magnitude of SPN in response to faces, such that children with lower T-scores (and thus less severe social impairments as reported by caregivers), showed larger SPNs in response to faces, *F*(1, 12) = 6.95, *p* = .021, Cohen's f^2^ = .577. *Figure 4* shows a scatter-plot of SRS-2 scores and amplitude in the face condition. However, it is can be noted that one subject elicited a particularly large SPN response, and thus may be considered an outlier, and when this subject was removed, the correlation no longer reached statistical significance, *F*(1,11) = 1.5, *n.s*.

## Discussion

### ERP

#### SPN

The current study suggests that there is not a significant difference in anticipation of a familiar versus an unfamiliar face for either children with ASD or their TD peers. However, TD children showed differences between conditions such that familiar faces elicited larger SPN compared to either of the arrow conditions, whereas unfamiliar faces were numerically larger (but not significantly different from) either arrow condition. This suggests that for TD children between the ages of 6–11 years old, familiar faces elicit a larger reward anticipation response compared to non-face stimuli. For children with ASD, we did not find any significant differences between conditions. Because we did not find the expected familiarity differences, we also explored whether the use of one repeated stimulus in each block would lead to habituation effects in either or both groups. We found a marginal effect of time, such that the first half of trials in each block elicited larger SPN responses than the second half, regardless of stimulus type or group. This suggests that although there is likely some habituation in the SPN response to a large number of repetitions of a single stimulus, it does not differ between groups or social versus nonsocial stimuli. Thus, it is unlikely that differences in the SPN response observed between groups are due to differences in how children with and without ASD habituate to stimuli, although habituation effects may explain the lack of familiarity effects in the present study.

Our results differ from several previous investigations [13], [17], [18], [22], [24], [25]. Key differences in our task compared to previous studies may explain this. Whereas previous studies have utilized passive viewing tasks, or tasks in which participants attend directly to images and respond to a target stimulus, the current study was designed such that pictures of faces (and scrambled versions of those images) were incidental to the task. In other words, participants did not need to attend to the face or arrow stimulus in order to gain information about whether their responses were “correct” or “incorrect.” Although this paradigm allowed us to directly control for physical stimulus properties and tangibility between conditions, it is difficult to directly compare our results with those found in previous studies.

In previous research, one group of authors found that children with ASD showed differential neural activity in response to familiar versus unfamiliar faces [13], and another group of authors found that a small subset of children with ASD began to show differential neural activity in response to familiar face after social skills training [17]. One potential reason for this discrepancy in previous literature may be due to stimulus differences between studies. Previous studies used multiple familiar and unfamiliar faces (rather than just one familiar and one unfamiliar face) [13]. With the exception of [17], which investigated neural activation after social skills training, Pierce and Redcay [13] was the only study to find differences between familiar and unfamiliar faces in children with ASD. One possibility is that children with ASD are more likely to differentiate between familiar versus unfamiliar faces when viewing multiple exemplars from each category. The finding in the current study that there was a marginal tendency for children across groups to habituate to the repeated presentation of a single stimulus supports this idea. Previous research suggests the fusiform face area (FFA) may be involved in determining the identity of individual faces [49]—thus, presenting multiple different faces may activate the FFA to a greater degree than presentations of single faces. It is possible that in previous research, presentation of multiple different familiar faces was adequate to normalize brain responses to faces in ASD. This is an interesting direction for future research, and future studies may wish to compare within subjects whether children with ASD elicit differential neural activity when viewing multiple faces versus one face.

Importantly, although we did not find a main effect of familiarity or interactions between group and familiarity, when we collapsed across familiarity for both groups, we found a group by condition interaction such that TD children showed a larger SPN component in response to faces versus arrows, while children with ASD demonstrated the opposite pattern. This replicates our previous work [29] with a novel group of participants and novel stimuli. These results are in line with the social motivation hypothesis—that TD children are more rewarded by social versus nonsocial stimuli, while children with ASD do not demonstrate this pattern.

Our results are consistent with previous studies that examined reward anticipation in these populations [27], [28], in that we found TD children and those with ASD elicited a statistically equivalent SPN response to *non*social feedback. Similarly, while the current study investigated reward anticipation of social versus nonsocial stimuli, and other ERP studies of the reward system in ASD have focused on reward processing of monetary stimuli only [30], [31], our results are consistent with these investigations insofar as we found that children with ASD elicit similar reward anticipation to their TD peers for nonsocial stimuli. Our results differ with regards to TD children, however, because we found that TD children elicited a larger SPN response to social versus nonsocial stimuli, whereas [28] found the opposite pattern. Our results also differ from behavioral measures of response inhibition for social versus monetary rewards [26], as those authors found that both TD children and those with ASD have increased performance for all reward types. However, the authors also found no difference in performance for familiar versus unfamiliar social stimuli in either group, which is consistent with the current findings [26].

One important difference between our current and previous findings is that current pairwise comparisons did not reveal a significant difference between the ASD and TD [29] groups for face stimuli. That is, while TD children had a significantly larger SPN to faces versus non-faces, there was not a significant difference between TD children and those with ASD for the face stimuli. This differs from our previous findings, where in addition to differences between face and non-face stimuli, TD children also had larger SPN responses to faces than children with ASD. One potential reason for this is stimulus variation. In our previous study, children saw a variety of unfamiliar faces, whereas in the current study they saw just one unfamiliar and one familiar face. When comparing our current results to our previous findings, TD children have a smaller SPN response in the face condition, while children with ASD have a larger SPN response in the face condition. In contrast, for the arrow condition, both groups are largely unchanged between studies. This raises the possibility that while TD children show larger SPN responses when viewing multiple faces, children with ASD demonstrate the opposite pattern. The current study was not designed to investigate this, and thus these possibilities remain conjecture, but future studies could manipulate the number of faces in the stimulus set, and measure resulting effects on the SPN.

#### Nc

We found an Nc-like component after participant's response, but before feedback. This component differentiated TD children from those with ASD. The component occurred at about the time (∼400 msec after the participant's button press) and had a similar scalp distribution (prominent at frontal electrode sites) as the Nc component that has typically been investigated in response to visual stimuli [50]. These findings provide novel information about the Nc component—in effect that the Nc can act as an anticipatory waveform. Previous findings have examined the Nc as a component related to salience and attention in response to a stimulus in infants and young children (e.g. [25]). Our findings, however, suggest that the Nc is also sensitive to anticipation of upcoming stimuli and/or the testing context (i.e., blocks of familiar and unfamiliar faces vs. arrows), and differentiates between diagnostic groups. It is important to note, however, that the current study was not designed to investigate anticipatory effects of the Nc component, as most studies on the Nc do not involve overt responses by the participant. Thus, while our results have interesting implications for the Nc, it is necessary for future studies to look directly at the effect of anticipation on the Nc between children with and without ASD.

### Brain and Behavior Correlations

The present results provide evidence that magnitude of reward anticipation response to faces in children with ASD can be predicted by reported levels of social impairments (as measured by the social responsiveness scales). This provides evidence that is in line with the social motivation hypothesis, insofar as children with lower levels of reported social impairments showed larger reward anticipation responses to faces compared to children with higher levels of reported impairments. We note, however, that this effect may have been driven by a single participant in the current study, so it is not advisable to draw large-scale conclusions from this analysis. Future studies should look into these types of correlations with a larger sample of children with ASD.

The current study has some limitations that should be noted. First, our sample size (*N* = 14 in each of the TD and ASD groups) is relatively small (although within the estimates provided by our power analysis). This makes it difficult to draw broad and generalized inferences. Further, we did not obtain information about treatment history from participants. Given previous findings about the effect of social skills training on face processing [17], as well as parent attitudes towards reward contingencies on behavioral sensitivity to rewards [26], this limitation should be taken into consideration when interpreting the current findings.

### Conclusions and Broader Implications

We examined reward anticipation of incidental familiar versus unfamiliar faces and scrambled versions of those images in children with and without ASD. Although we did not find evidence for an effect of familiar versus unfamiliar faces in either group, the current study adds to the body of literature supporting the social motivation hypothesis, and replicates previous findings using different stimuli and participants. The current study also provides evidence that magnitude of reward anticipation to faces is significantly correlated with levels of parent-reported social impairments. This suggests that our paradigm is sensitive to social impairments as measured by questionnaires, which provides evidence that we are accurately capturing social motivation in children with ASD.

Our findings provide interesting implications for future work on the Nc-like component, which we observed as a measure of anticipation in children, and suggest that for TD children, anticipation of face stimuli elicits a larger Nc-like component than for children with ASD. While our study was not designed to directly address this question, we feel it is an important future direction. The current study also suggests intriguing areas for future research in regards to whether children with and without ASD are differentially affected by viewing one versus multiple unfamiliar faces. The current study and previous work suggest that perhaps TD children show larger reward anticipation for multiple unfamiliar faces, while children with ASD show the opposite pattern. However, because the current and previous studies utilized different participants and stimuli, we suggest this as an important future direction.

The current study suggests that social motivation deficits in ASD are not ameliorated by viewing familiar faces when face stimuli are incidental to the task. Future research is necessary to determine whether task specifications or number of faces within a stimulus set affects these findings. The current study provides further evidence for the social motivation hypothesis, and suggests that levels of social impairment in ASD are correlated with magnitude of reward anticipation to faces. This paradigm could be utilized as a biomarker of social motivation, and could be used before and after behavioral or pharmacological interventions designed to improve social motivation. In this way, individual children's levels of reward anticipation to faces could be tracked over time along with behavioral levels of social impairment, in order to see changes throughout the course of intervention.
